# Longitudinal measurement invariance of the Child Problematic Trait Inventory in older Chinese children

**DOI:** 10.1371/journal.pone.0219136

**Published:** 2019-07-03

**Authors:** Jie Luo, Xuetong Wang, Meng-Cheng Wang, Xintong Zhang, Jiaxin Deng, Chuxian Zhong, Yu Gao, Shi-san Qi

**Affiliations:** 1 School of Psychology, Guizhou Normal University, Guiyang, China; 2 School of Psychology, Inner Mongolia Normal University, Hohhot, China; 3 The Center for Psychometrics and Latent Variable Modeling, Guangzhou University, Guangzhou, China; 4 School of Management, Guangzhou University, Guangzhou, China; 5 Department of Psychology, Guangzhou University, Guangzhou, China; 6 The Key Laboratory for Juveniles Mental Health and Educational Neuroscience in Guangdong Province, Guangzhou University, Guangzhou, China; 7 Brooklyn College, the City University of New York, Brooklyn, New York, United States of America; University of Lleida, SPAIN

## Abstract

The Child Problematic Traits Inventory (CPTI) is a newly developed informant-rated instrument to measure psychopathic traits during early childhood. The aim of this study was to examine the longitudinal measurement invariance of the CPTI in a group of Chinese schoolchildren. Mothers of 585 children aged 8 to 12 years (50% girls) completed the CPTI twice with one-year interval. Confirmatory factor analyses showed that the CPTI had strict invariance (i.e., equality of factor patterns, loadings, intercepts, and item uniqueness) across time. Furthermore, the internal consistencies for the CPTI subscales were good at both time points and the stability coefficients over time were moderate. Findings suggest that, in children aged 8 to 12 years old, changes in CPTI scores across time can be attributed to actual changes in the child’s psychopathic personality.

## Introduction

Psychopathy or psychopathic traits has been a common area of research in psychopathology and psychology [1, 2]. Psychopathic traits are traditionally described as a syndrome comprising a constellation of concurrent personality traits being captured under at least three dimensions: interpersonal (e.g., grandiosity, deceitfulness), affective (e.g., callousness, lack of empathy), and lifestyle (e.g., impulsivity, need for stimulation) [3–5]. Psychopathic traits have been linked to severe conduct problems, long-lasting psychosocial problems, delinquency, behavior maladjustment, and various forms of aggressive behavior [6–10]. It is believed that psychopathic traits do not emerge suddenly in early adulthood, but rather that their roots may lie in childhood and adolescence [10–13]. Therefore, understanding the development and variation of psychopathic traits from childhood into adolescence and adulthood is crucial for understanding the development and stability of serious and persistent conduct problems and criminal behavior [6, 7, 9]. Indeed, investigating psychopathic personality in children and adolescents might help psychologist and psychopathologist to understand the etiology of adult psychopathy, and provide preventive interventions or early treatment programs [14, 15]. It is thus that an increasing number of studies focuses on psychopathic personality in children and adolescents [15, 16].

Extensive studies have shown that psychopathic traits can be measured reliably during both childhood and adolescence [17–20], and that the results are moderately stable over time [21–23]. With respect to the psychopathy construct, several studies have indicated that psychopathy in children and adolescents have been demonstrated to combine into the same three dimensions that comprise adult psychopathic traits [19, 20, 24, 25]. To increase the possibility of evaluating the construct, stability and development of psychopathic traits between childhood (including early childhood) and adolescence or adulthood, an excellent instrument must be developed to assess psychopathic traits or the psychopathy construct as needed [8, 9, 26]. Especially, to investigate and understand when psychopathic traits develop and how early in life they can be detected and measured in a meaningful way.

The present investigation will thus focus on the Child Problematic Traits Inventory (CPTI) [9], a new instrument that has been designed to evaluate the psychopathic traits in children between the ages of 3 and 12, as well the CPTI’s ability to enable longitudinal studies that test development and stability of psychopathic traits across different developmental phases.

### The Child Problematic Trait Inventory (CPTI)

The CPTI is a newly-developed teacher-rated instrument used to measure psychopathic traits in early childhood [9]. It was developed to assess the construct of psychopathic personality in childhood—including in early childhood—and is designed to closely resemble how traits are usually conceptualized and assessed in adolescence and adulthood, often with interpersonal, affective, and lifestyle components [3, 9, 19].

Two fundamental principles guided and assisted in the selection of traits and items to be considered in the CPTI. First, only traits that were within the framework of the three-factor solution conceptualization of psychopathic traits that have theoretical and/or empirical support for being evaluable and applicable in children between the ages of 3 and 12 should be included. Second, traits that are closely related to or even overlap conceptually with conduct problems and antisocial behavior should be excluded. This was done to avoid issues with contamination when using the CPTI as a measure of psychopathic traits in research that aimed to understand the development of conduct problems and antisocial behavior [9, 27].

On the basis of a theory-driven approach and factor analysis for the CPTI, 28 items were included. Teachers rated the frequency of each item on a four-point Likert scale (1 = *does not apply at all*; 2 = *does not apply well*; 3 = *applies fairly well*; and 4 = *applies very well*). These 28 items were intended to load on three theoretically proposed dimensions: interpersonal (Grandiose–Deceitful; eight items), affective (Callous-Unemotional; 10 items), and behavioral (Impulsive–Need for Stimulation; 10 items). Furthermore, these three factors were assumed to load onto an overarching latent factor (i.e., psychopathic traits).

According to Colins and colleagues (2014) [9], both exploratory and confirmatory factor analyses suggested that the proposed three-factor model fit the data well overall for children aged 3 to 5 years (mean age = 3.86; *SD* = .86; *N* = 2,056; 53% male; CFI = .96, TLI = .96, RMSEA = .07), for both genders (boys / girls: CFI = .97 / .96; TLI = .96 / .96; RMSEA = .07 / .07, respectively), and across all ages (3- / 4- / 5-year-olds: CFI = .98 / .97 / .97; TLI = .97 / .96 / .97; RMSEA = .07 / .07 / .07, respectively). The CPTI scores also showed good to excellent internal consistencies in the overall sample for each gender group and across all ages (αs from .88 to .96). As for external validity, the three factors were positively associated with conduct problems, symptoms of attention-deficit / hyperactivity disorder (ADHD), and fearlessness, and they negatively correlated with an easy temperament. These correlations were also significant when controlling for multiple sociodemographic variables, including age, socioeconomic status, ethnicity, and gender [9]. In sum, the original CPTI holds promise as a reliable and valid tool to measure a constellation of traits in young children that is similar to a psychopathic personality as it manifests in adolescents and adults.

To our knowledge, following the original Swedish investigation of the CPTI [9], five published studies to date have replicated and extended the psychometric support for the CPTI in four languages other than English (i.e., Dutch [28]; Italian [29]; Chinese [26]; and Spanish [30]). Each version of the CPTI has good to excellent psychometric properties, indicating that it is a promising tool for assessing psychopathic traits in children across different cultures (i.e., both Western and Chinese samples) [26, 28–31] (see Table 1).

**Table 1:** Summary of study characteristics and psychometric properties in prior studies of the CPTI.

Authors	Form	Sample characteristics	Country	Method	Best model	Alpha (number of items)	Fit indices
Colins et al. (2016)	TR	1,188 children: 50.3% female, aged 5 years	Sweden	CFA: WLSMV	Three-factor model	***Total sample*** Total .96(28), GD .90(8), CU .94(10), INS .91(10)	***Total sample*** CFI = .95, TLI = .94, RMSEA = .08
						***Male*** Total .96(28), GD .89(8), CU .95(10), INS .91(10)	***Male*** CFI = .95, TLI = .95, RMSEA = .08
						***Female*** Total .95(28), GD .91(8), CU .93(10), INS .91(10)	***Female*** CFI = .95, TLI = .95, RMSEA = .07
Somma et al. (2016)	TR; FR; MR	***Sample one*** 381 children: 52.2% female, aged 6 to 12 years; *M* = 8.78, *SD* = 1.64	Italy	CFA: WLSMV	Three-factor model	***Sample one*** **TR**: Total .95(28), GD .90(8), CU .94(10), INS .89(10) **MR**: Total .92(28), GD .85(8), CU .86(10), INS .85(10) **FR**: Total .92(28), GD .85(8), CU .88(10), INS .84(10)	***Sample one*** **TR**: CFI = .958, TLI = .954, RMSEA = .078 **MR**: CFI = .947, TLI = .941, RMSEA = .057 **FR**: CFI = .942, TLI = .937, RMSEA = .061
		***Sample two*** 406 children: 51.5% female, aged 6 to 12 years; *M* = 8.49, *SD* = 1.56				***Sample two*** **TR**: Total .96(28), GD .93(8), CU .93(10), INS .93(10) **MR**: Total .92(28), GD .85(8), CU .88(10), INS .84(10) **FR**: Total .92(28), GD .86(8), CU .88(10), INS .85(10)	***Sample two*** **TR**: CFI = .966, TLI = .963, RMSEA = .078 **MR**: CFI = .950, TLI = .945, RMSEA = .059 **FR**: CFI = .944, TLI = .938, RMSEA = .064
Colins et al. (2018)	TR	287 children: 48% female, aged 3 to 7 years; *M* = 4.35, *SD* = .88	Dutch	CFA: WLSMV	Three-factor model	Total .96(28), GD .92(8), CU .95(10), INS .91(10)	CFI = .97, TLI = .97, RMSEA = .079
López-Romero et al. (2018)	TR	***Sample one*** 449 children: 51.4% female, aged 3 to 12 years; *M* = 7.32, *SD* = 2.69 ***Sample two*** 393 children: 1.1% female, aged 3 to 12 years; *M* = 7.82, *SD* = 2.57	Spain	CFA: WLSMV	Three-factor model	***Total sample*** Total .97(28), GD .93(8), CU .95(10), INS .93(10)	***Total sample*** CFI = .97, TLI = .97, RMSEA = .08 ***Sample one*** CFI = .96, TLI = .96, RMSEA = .10 ***Sample two*** CFI = .98, TLI = .97, RMSEA = .06
Wang et al. (2018)	TR; MR	686 children: 48.1% female, aged 6 to 12 years	China	CFA: WLSMV	Three-factor model	***Total sample*** **TR**: Total .95(28), GD .87(8), CU .92(10), INS .88(10) **MR**: Total .92(28), GD .79(8), CU .86(10), INS .81(10)	***Total sample*** **TR**: CFI = .94, TLI = .93, RMSEA = .09 **MR**: CFI = .92, TLI = .91, RMSEA = .07.
						***Male*** **TR**: Total .95(28), GD .89(8), CU .91(10), INS .86(10) **MR**: Total .92(28), GD .81(8), CU .87(10), INS .83(10)	
						***Female*** **TR**: Total .95(28), GD .83(8), CU .90(10), INS .89(10) **MR**: Total .90(28), GD .76(8), CU .84(10), INS .80(10)	

TR = Teacher-rated; MR = Mother-rated; FR = Father-rated; CFA = Confirmatory Factor Analysis; WLSMV = Robust Weighted Least Squares with Mean and Variance Adjustment; CFI = Comparative Fit Index; TLI = Tucker-Lewis Index; RMSEA = Root Mean Square Error of Approximation; GD = Grandiose–Deceitful; CU = Callous–Unemotional; INS = Impulsive–Need for Stimulation.

Three out of the four studies mentioned above (e.g., Dutch [28]; Spanish [30]; Swedish [31]) relied on teacher-rated CPTI data and the Italian version of the CPTI were examined using data from teachers, mothers, and fathers [29]. All the four studies supported the original three-factor structure (i.e., Dutch: CFI = .97, TLI = .97, and RMSEA = .08; Spanish: CFI = .97, TLI = .97, and RMSEA = .08; Swedish: CFI = .95, TLI = .94, and RMSEA = .08; Italian: CFI from .94 to .97; TLI from .94 to .96; RMSEA from .06 to .08). These CPTI scores also showed good to excellent internal consistencies (e.g., Dutch: αs from .91 to .96, and MICs from .47 to .67; Spanish: αs > .90, MICs > .50; Swedish: αs from .89 to .96; Italian: αs from .84 to .96), as well as adequate external validity by mean of the expected correlations with conduct problems, aggression and temperament [28–31].

Recently, Wang and colleagues (2018) investigated the psychometric properties of the teacher- and mother-rated CPTI in 6- to 12-year-old Chinese children [26]. In line with prior studies, the three-factor structure was supported using each of the two informants (i.e., teacher- / mother-reported: RMSEA = .09 / .07, CFI = .94 / .92, and TLI = .93 / .91), although the RMSEA for the teacher version was above the commonly used value of .08. The internal consistencies of the CPTI total and factor scores were satisfactory in both groups (i.e., boys and girls) and across informant (e.g., αs from .76 to .95, MICs from .26 to .52). Moreover, the teacher- and mother-reported CPTI scores showed expected relations with conduct problems, hyperactivity, and various dimensions of temperament (Wang et al., 2018). Finally, according to Wang et al. (2018), the teacher’s rated CPTI scores were invariant across gender while the mother rated version was not invariant over gender [26].

In general, the cross-sectional data [9, 26, 28–31] supports the proposed psychometric properties of the CPTI. Yet, none of the studies have focused on the longitudinal properties of the CPTI and, in particular, the longitudinal measurement invariance of the CPTI across different occasions.

### Measurement invariance of the CPTI

Measurement invariance is important because the interpretation of mean differences could be misleading or problematic unless the underlying constructs are the same across different groups [32, 33]. In other words, the establishment of measurement invariance is a prerequisite for making meaningful comparisons across groups (e.g., girls vs. boys) [33].

Prior studies have explored measurement invariance of the CPTI for gender and grade [26, 30]. For example, Wang and colleagues (2018) found that the teacher-rated CPTI has the property of strict invariance across gender in 6- to 12-year-old Chinese children [26]. Existing studies have largely only focused on measurement invariance across gender [26], giving no understanding of whether the longitudinal measurement invariance (LMI; i.e., measurement invariance across different times) of the CPTI has been obtained.

LMI, which assumes that the same construct of the instrument is assessed across time periods, is an important factor that must be addressed to ensure the validity of mean comparison in longitudinal studies [34, 35]. Similar to measurement invariance between gender groups, the LMI examines the equality of the factor structure for an instrument, yet its focus is on equality across time [36, 37]. Furthermore, the LMI has two unique characteristics. First, the latent constructs across time can be correlated with each other in the longitudinal structure. Second, the corresponding unique factors for each indicator are allowed to covary across occasions. More specifically, LMI is a desirable measurement to have because it assumes that the same construct can be assessed across time (i.e., the configural, metric, scalar, and residual invariance), providing a solid basis for mean comparisons. Without the premise of LMI, any inference about developmental changes across time could be misleading or inaccurate [35–37]. The current study was the first to examine whether the CPTI has LMI across different time measurements.

### The present study

The primary aim of the current study was to test the LMI of the CPTI in a sample of 8- to 12-year-old schoolchildren in Mainland China. To this end, a series of confirmatory factor analyses (CFAs) were conducted, and we expected that the original three-factor solution of the CPTI would fit the data well at each time point. More importantly, additional CFAs would be conducted to examine whether the CPTI scores had LMI across time. Specially, we would test the configural, metric, scalar, and residual invariance over a one-year interval. It was expected that the CPTI would have a strict invariance property (i.e., an equality of factor patterns, loadings, intercepts, and item uniqueness). Furthermore, the internal consistency index of the total and subscale scores would be examined at each measured time point. Finally, the factor scores at two time points would be compared, and their stability across time would be investigated.

## Materials and methods

### Participants

The data for the present study was collected from a primary school in Guangdong, China. Using this ongoing, longitudinal study, we aim to further understand the correlates and causes of heterogeneity in children’s behavior, social adjustment, and psychological health. Parts of the data have been previously reported in Wang and colleagues (2018) using the first-wave data collected in 2015, while the present study reports the fourth- and fifth-wave data, collected in 2016 and 2017, respectively.

The first survey was administered in October 2016 (Time 1). Mothers of 613 children completed the Chinese version of the 28-item three-factor CPTI [26]. One year later (Time 2), all first-wave participants were invited to take part in a follow-up assessment, and mothers of 585 children participated. An independent-samples *t-*test was performed to ascertain whether the attrition at Time 2 was random. Results revealed that the three factors and total scores of the CPTI at Time 1 were not significantly different between those who did and who didn’t participate at Time 2 (e.g., GD: *t* = -.283, *p* = .777; CU: *t* = -.588, *p* = .557; INS: *t* = -.315, *p* = .753; CPTI total: *t* = -.823, *p* = .411), indicating that the sample attrition at Time 2 was random. Of the final sample, 292 (49.9%) were boys and 293 (50.1%) were girls, with 138 (23.6%), 163 (27.9%), 123 (21.0%), and 161 (27.5%) in the 3^rd^, 4^th^, 5^th^, and 6^th^ grade, respectively, at Time 1. Information regarding the children’s number of siblings, family composition, education level, and parental monthly income is presented in Table 2.

**Table 2:** Demographic information for the present sample.

Variables	Number (%)
Number of siblings	
None	347 (59.3%)
One or more	238 (40.7%)
Family composition	
Mother and father	427 (73.0%)
Single parent	20 (3.4%)
Missing information	138 (23.6%)
Educational level [mother/father]	
Primary school or below	22 (3.8%) / 20 (3.4%)
Junior middle school	50 (8.5%) / 39 (6.7%)
Senior middle school	109 (18.6%) / 97 (16.6%)
Bachelor degree or above	240 (41.0%) / 259 (44.3%)
Missing information	164 (28.0%) / 170 (29.1%)
Monthly income ^a^	
< 4,000	72 (12.3%)
4,000–8,000	236 (40.3%)
8,000–12,000	159 (27.2%)
12,000–16,000	57 (9.7%)
16,000–20,000	28 (4.8%)
> 20,000	23 (3.9%)
Missing information	10 (1.7%)

^a^ The monetary unit of income is Yuan.

### Measures

#### The Child Problematic Traits Inventory (CPTI)

As described in detail previously [9, 26], the CPTI has 28 items and each item is rated on a four-point Likert scale (1 = *does not apply at all*, 2 = *does not apply well*, 3 = *applies fairly well*, 4 = *applies very well*). In addition to the three-factor scores (GD, eight items; CU, 10 items; and INS, 10 items), the CPTI yields a total score to measure the subject’s general level of psychopathy. The higher the CPTI scores, the higher the psychopathic level [9]. All CPTI items are displayed in Table 3. In the present sample, the alpha coefficients for the GD, CU, and INS factors at the two time points measured were .83 (MIC = .39) /.86 (MIC = .44), .89 (MIC = .45) / .91 (MIC = .51), and .86 (MIC = .38) /.88 (MIC = .43), respectively.

**Table 3:** Descriptive statistics for the CPTI at two time points.

Item	Time 1 CPTI				Time 2 CPTI			
	*M*	*SD*	*SK*	*KU*	*M*	*SD*	*KU*	*SK*
5—Lies often to avoid…	1.93	.76	.44	-.27	1.85	.77	.69	.15
7—Seems to see himself…	2.23	.72	.18	-.18	2.22	.75	.24	-.19
9—Often lies to get what…	1.68	.71	.83	.50	1.57	.70	1.05	.73
15—Seems to lie more…	1.51	.69	1.27	1.32	1.45	.66	1.42	1.70
18—Is often superior…	1.48	.64	1.19	1.11	1.44	.62	1.29	1.63
21—To get people to do…	1.49	.69	1.27	1.15	1.44	.68	1.49	1.85
24—Think that he/she is…	2.00	.65	.19	-.05	1.96	.69	.22	-.40
26—To frequently lie…	1.37	.61	1.59	2.20	1.38	.63	1.64	2.33
2—Seldom express…	1.71	.69	.64	.01	1.70	.75	.96	.67
4—Usually does not…	1.80	.64	.37	.07	1.74	.67	.64	.43
8—Never seems to have…	1.88	.68	.55	.56	1.87	.67	.57	.78
11—Often seems to be…	1.57	.63	.90	.93	1.54	.63	.91	.57
13—Does not become…	1.60	.64	.82	.76	1.56	.67	.93	.25
17—Seldom remorseful…	1.90	.69	.35	-.10	1.89	.71	.31	-.53
20—Often does not seem…	1.80	.70	.55	.04	1.74	.70	.61	.00
22—Sometimes seems to…	1.81	.69	.39	-.36	1.81	.72	.42	-.61
25—Never expresses…	1.85	.69	.39	-.21	1.77	.69	.50	-.24
27—Does not express…	1.64	.65	.67	.12	1.68	.70	.66	-.31
1—Likes change and…	2.26	.68	.24	.04	2.03	.65	.35	.51
3—Often has difficulties…	2.41	.75	.03	-.33	2.09	.73	.15	-.46
6—Seems to do certain…	2.27	.75	.13	-.31	2.17	.77	.08	-.63
10—Provides himself or…	1.91	.77	.50	-.23	1.78	.76	.72	.06
12—Often does things…	2.05	.79	.33	-.43	1.83	.79	.69	-.06
14—Often consumes things…	1.93	.79	.54	-.17	1.89	.80	.59	-.21
16—Seems to have a…	2.05	.74	.40	-.00	1.98	.77	.47	-.10
19—Does not like waiting…	2.28	.79	.02	-.59	2.05	.78	.21	-.66
23—Seems to get bored…	2.03	.78	.32	-.46	1.93	.78	.43	-.46
28—Quickly gets tired of…	1.89	.83	.71	-.06	1.80	.81	.71	-.25

CPTI = Child Problematic Traits Inventory; SK = Skewness; KU = Kurtosis.

### Procedures

The head of each school and the students’ teachers were informed about the purposes of the study. Parental information, consent forms, and questionnaires were enclosed in envelopes and sent home with the children. The parents were asked to complete the questionnaires at home, and then to return the questionnaires in a sealed envelope to their child’s teacher within two days. In the present study, all questionnaires were completed at home by each subject’s mother. The study was reviewed and approved by the Human Subjects Review Committee at Guangzhou University (Review NO.20141008).

### Data analysis

First, descriptive statistical analysis was performed with SPSS 22.0 [38]. Next, a series of confirmatory factor analyses (CFAs) were conducted with Mplus 7.0 [39] to test the LMI across the measurement points. The proposed three-factor solution was considered as the baseline model. In this model, the 28 items were loaded on the three factors (i.e., GD: eight items; CU: 10 items; INS: 10 items) at each time point. The model fit is considered acceptable if both the comparative fit index (CFI) and the Tucker–Lewis index (TLI) are higher than .90, and the root mean square error of approximation (RMSEA) is lower than .80. The model fit is considered good if both the CFI and TLI are higher than .95 and the RMSEA value is lower than .05 [40]. Given cutoff standards may be specific to particular models and data sets [41], we adopt multiple indices to choose model. Specifically, when fit indices give inconsistent conclusion, we will take majority rule.

The items of the CPTI have only four response categories, specifically, measured ordered-categories. Because simulation studies have shown that a minimum of five response categories is a prerequisite for the assumption of continuity underlying the maximum likelihood (ML) estimation [42–44], the ML estimation was deemed inappropriate. Therefore, the robust weighted least squares with mean and variance adjustment (WLSMV) estimator was used in the current study [45, 46].

Next, tests for the LMI were conducted with four nested models by successively setting the equality of the parameters of the measurement model across occasions. In general, the procedure for testing the LMI with categorical data closely parallels that with continuous data. Specifically, the configural invariance tests the hypothesis that the same general pattern of factor loadings (e.g., two-factor vs. three-factor) holds across occasions [37, 46]; the metric invariance requires the corresponding factor loadings across time to be equal; the scalar invariance sets the corresponding factor loadings and intercepts to be equal across occasions; and the residual variance invariance requires the corresponding factor loadings, intercepts, and residual variances of items across time to be equal. To compare nested models, change in CFI (ΔCFI) was used, with changes smaller than .01 indicating equality between the more restricted model and the less restricted model [47, 48]. Also, as recommended by Chen (2007), a change in RMSEA (ΔRMSEA) higher than .015 suggests an absence of invariance [48]. The chi-square difference test (using the DIFFTEST option in Mplus) was not used due to its sensitivity to minor parameter changes in large samples [48].

The internal consistency values of the CPTI at each time point were then examined. Alpha coefficients below .60 are considered insufficient, between .60 and .69 are marginal, .70 to .79 are acceptable, between .80 and .89 are good, and above .90 are excellent [49]. Furthermore, we inspected the means inter-item correlations (MIC) that are independent of scale lengths; MICs in the range of .15 to .50 are considered acceptable [50].

Next, the stability coefficients (i.e., correlations between the CPTI factor scores at two time points) were computed using the strict invariance model to examine whether the psychopathic personality is moderately stable [23]. In order to compute the factor correlations, factor variances are set to one, and the first factor loading for each factor is freely estimated.

Finally, the means of the latent factors were compared between the two time points to explore the development of the psychopathic personality. To do this, we set the three factor means at Time 1 to zero and freely estimate the factor means at Time 2.

## Result

### Descriptive statistics

Descriptive statistics for each item over time are presented in Table 3, including the means, standard deviations, skewness, and kurtosis.

### LMI of the CPTI

The LMI analysis for the CPTI was conducted in the following way. Model fits are shown in Table 4. First, at each time point, the proposed three-factor model fit the data adequately, allowing for further examinations of the LMI. Additionally, the baseline model of configural invariance is acceptable (i.e., CFI = .935; TLI = .931; RMSEA = .052).

**Table 4:** Fit indices for longitudinal measurement invariance.

Model	WLSMV χ2	*df*	CFI	TLI	RMSEA (90% CI)	Δχ2 (*p*)	ΔCFI	ΔTLI	ΔRMSEA
Time 1	1748.473***	347	.924	.918	.083 (.079, .087)				
Time 2	1716.225***	347	.932	.926	.082 (.078, .086)				
Configural	3700.525***	1444	.935	.931	.052 (.050, .054)	—	—	—	—
Metric	3734.606***	1469	.935	.932	.051 (.049, .053)	70.789 ***	0	.001	-.001
Scalar	3747.948***	1522	.936	.935	.050 (.048, .052)	79.365*	.001	.003	-.001
Residual	3564.335***	1550	.942	.943	.047 (.045, .049)	67.966***	.006	.008	-.003

WLSMV χ2 = Weighted Least Squares with Mean and Variance Adjusted Chi-square; *df* = Degrees of Freedom; CFI = Comparative Fit Index; TLI = Tucker-Lewis Index; RMSEA = Root Mean Square Error of Approximation; 90% CI = 90% Confidence Interval around RMSEA; Δχ^2^ = Change in χ^2^ Relative to the Preceding Model; ΔCFI = Change in Comparative Fit Index Relative to the Preceding Model; ΔTLI = Change in Tucker-Lewis Index Relative to the Preceding Model; ΔRMSEA = Change in Root Mean Square Error of Approximation Relative to the Preceding Model;

* *p* < .05,

** *p* < .01,

*** p < .001.

Next, the factor loadings were constrained to be equal across time to test for the metric invariance. The metric invariance model fit was adequate (i.e., CFI = .935; TLI = .932; RMSEA = .051), and the differences of CFI, TLI, and RMSEA between the configural and metric invariance models are negligible (i.e., ΔCFI = 0; ΔTLI = .001; ΔRMSEA = -.001).

We then tested the scalar invariance model in which all item intercepts were restricted to be equal across time. The scalar invariance model provided a satisfactory fit (i.e., CFI = .936; TLI = .935; RMSEA = .050), and again the changes in the CFI, TLI, and RMSEA were negligible (i.e., ΔCFI = .001; ΔTLI = .003; ΔRMSEA = -.001). Finally, equality of item uniqueness across occasions was imposed on the model to test for residual invariance. The residual invariance model fit the data adequately (i.e., CFI = .942; TLI = .943; RMSEA = .047) with negligible differences in the CFI, TLI, and RMSEA between the strong and strict invariance models (i.e., ΔCFI = .006; ΔTLI = .008; ΔRMSEA = -.003). The residual invariance of the CPTI scores across time was therefore supported. Overall, our results suggested that the three-factor solution of the CPTI had LMI over a one-year period. The standardized factor loadings of the longitudinal factor model are presented in Table 5.

**Table 5:** Standardized factor loadings for the longitudinal factor model of CPTI.

Item	Time 1			Time 2		
	GD1	CU1	INS1	GD2	CU2	INS2
5—Lies often to avoid…	.754			.841		
7—Seems to see himself…	.305			.398		
9—Often lies to get what…	.707			.805		
15—Seems to lie more…	.846			.907		
18—Is often superior…	.728			.822		
21—To get people to do…	.878			.928		
24—Think that he/she is…	.462			.577		
26—To frequently lie…	.888			.934		
2—Seldom express…		.622			.669	
4—Usually does not…		.762			.800	
8—Never seems to have…		.707			.750	
11—Often seems to be…		.746			.786	
13—Does not become…		.716			.758	
17—Seldom remorseful…		.758			.797	
20—Often does not seem…		.831			.861	
22—Sometimes seems to…		.794			.829	
25—Never expresses…		.840			.869	
27—Does not express…		.813			.846	
1—Likes change and…			.453			.475
3—Often has difficulties…			.749			.769
6—Seems to do certain…			.707			.729
10—Provides himself or…			.698			.720
12—Often does things…			.776			.795
14—Often consumes things…			.674			.697
16—Seems to have a…			.609			.633
19—Does not like waiting…			.734			.755
23—Seems to get bored…			.798			.816
28—Quickly gets tired of…			.767			.786

CPTI = Child Problematic Traits Inventory; GD1 = Grandiose–Deceitful at Time 1; CU1 = Callous–Unemotional at Time 1; INS1 = Impulsive–Need for Stimulation at Time 1; GD2 = Grandiose–Deceitful at Time 2; CU2 = Callous–Unemotional at Time 2; INS2 = Impulsive–Need for Stimulation at Time 2; all factor loadings are significant at the level of *p* < .001.

### Internal consistency, stability coefficient, and latent mean comparisons

In terms of internal consistencies, the alpha coefficients for the three CPTI factors, as well as the CPTI total scores, were good (i.e., greater than .80) at each time point. For the GD factor, coefficient αs at Time 1 and Time 2 were .83 (MIC = .39) and .86 (MIC = .44), respectively. For the CU factor, coefficient αs were .89 (MIC = .45) and .91 (MIC = .51). For the INS factor, coefficient αs were .86 (MIC = .38) and .88 (MIC = .43). For the CPTI total score, coefficient αs were .93 (MIC = .33) at Time 1 and .95 (MIC = .41) at Time 2. Additionally, the stability coefficient (i.e., correlations between factor scores at the two time points) was computed using the strict invariance model. Results showed that the correlations between the GD, CU, and INS at Time 1 and Time 2 were .67, .57, and .65, respectively (*ps* < .001).

Given that the strict invariance model was supported, the means of the latent factors at the two time points could be meaningfully compared. The means of the three factors were significantly lower at Time 2 than at Time 1, with the following results: *t* = -.238, *p* < .001 for GD; *t* = -.086, *p* = .031 for CU; *t* = -.299, *p* < .001 for INS.

## Discussion

The purpose of the present study was to test the LMI of the CPTI, a newly-developed instrument designed to assess the Grandiose–Deceitful, Callous–Unemotional, and Impulsive–Need for Stimulation factors of psychopathic traits in children aged of 3 to 12 years old [9]. Our findings provide further support for the three-factor structure of the CPTI and, more importantly, show that this structure is invariant across a one-year time interval (i.e., equality of factor patterns, loadings, intercepts, and item uniqueness across time for all 28 items). Also, the internal consistency and stability coefficients support the stability of the CPTI scores over time. Overall, our findings replicated and extend previous work on the psychometric properties of the CPTI [9, 26, 28–31], and for the first time demonstrate that meaningful comparisons across time (specifically, a one-year period) can be conducted using the CPTI as it assesses the same construct at each different time point.

### Longitudinal measurement invariance of the CPTI

The LMI examines whether the same constructs are assessed across different time points within the same group to ensure that changes in test scores over time can be attributed to actual changes in the construct under investigation [36, 37, 46]. Although the psychometric properties of the CPTI had been supported in cross-sectional data [9, 26, 28–31], no study had yet examined the LMI of the CPTI. Indeed, if the LMI does not hold across time interval, any inference about the development and variation of psychopathic traits in observed in CPTI scores across different phases may not be meaningful and inaccurate. To address this gap, the current study examined the LMI of the CPTI in children aged 8 to 12 years old.

Our results demonstrate the strict longitudinal measurement variance [47, 48] (i.e., configural invariance: the same structure across time; metric invariance: the same factor loadings across occasions; scalar invariance: the same intercepts over time; residual variance invariance: the same error variance across time) of the CPTI over a one-year period, suggesting that the CPTI measured the same construct at different occasions, and the psychopathic traits concept in late children is to be stable over time [23]. Importantly, this implies that the mean difference in psychopathy scores as assessed by the CPTI across time could be interpreted as true changes in the level of psychopathic traits of an individual. Furthermore, these findings have significant implications for longitudinal studies using the CPTI. For example, in typical longitudinal models (e.g., latent growth model; LGM), the matrix of input becomes enormous, with data collected at multiple time points. To address this issue, item parceling is commonly used. The use of parcels as indicators, nonetheless, may mask the measurement invariance tests at the item parcel level [51]. Therefore, our finding of strict LMI of the CPTI at item level provides rationale for the use of item parcel sets in longitudinal models. Further, the LMI of the CPTI is particularly relevant for developmental psychologists and psychopathologists who are interested in the development of children psychopathy. As no prior CPTI study has formally and comprehensively tested the LMI of CPTI scores, future studies are needed to replicate and extend the current findings in other age categories (e.g., children aged 3 to 7 years old).

### Internal consistency and stability coefficients across time

Internal consistency coefficients across measured time points also provided some useful perspectives into the stability of the CPTI scores. Consistent with findings from cross-sectional studies [9, 26, 28–31] and one longitudinal study [52], the internal consistency coefficients for the CPTI subscale scores in the current sample were above .80 and, for the CPTI total scores, greater than .90 at both time points. Also, the MICs are high overall, and well above .30 for the CPTI. It may suggest that the traits that are applicable to children in line with the three dimensions of psychopathic personality, is still reliable across time. In general, our findings support that CPTI scores have satisfactory internal consistency across measured time points.

Additionally, the latent factor scores at Time 1 and Time 2 were significantly correlated (i.e., *r*s ranged from .57 to .67). Consistent with correlations at the observed variable level [21, 53], these strong correlations between latent factors suggest that the psychopathic traits are at least moderately stable over a one-year period [21, 23, 53]. Moreover, previous longitudinal studies [22] that have examined the relationship between psychopathy assessed in adolescence by using the mother-rated measures and psychopathy evaluated in adulthood using interviewed-reported scores, have shown that psychopathy from early adolescence into young adulthood was moderately stable [22]. In other words, psychopathy or a psychopathic personality is developmental, and stable traits can be identified in childhood, adolescence, and adulthood [22, 54]. Also, psychopathy as measured in adolescence is predictive of serious delinquency in later adolescence, and of psychopathy in adulthood [54]. It is therefore no surprising that psychopathic traits in children and adolescents might help researchers to gain and understand the etiology of adult psychopathic personality, and provide preventive interventions or early treatment programs [15, 16].

### Latent factor mean comparisons

Given that the LMI of the CPTI scores was supported, direct comparisons of the means of the latent factors can provide meaningful information. Consistent with prior longitudinal studies [21, 52], in the current study sample all three dimensions of the psychopathic traits were significantly reduced between Time 1 to Time 2. It may suggest that the level of psychopathic tendencies decreases as children grow. Additionally, previous prospective studies [55–57] have provided evidence of important individual differences in the early developmental course of psychopathic traits. More importantly, different developed trajectories of psychopathic traits (i.e., stable high, increasing, decreasing, and stable low) and two trajectories (i.e., high or low) of conduct problems (CP) were identified in childhood [55], as well as five trajectories (e.g., low, moderate, adolescent-onset, childhood-limited, and early-onset chronic) of IC across both childhood and adolescence [56]. Therefore, there is enough reason to believe that significant within-person heterogeneity exists in the developmental course of psychopathy during both childhood and adolescence [54–57]. However, it is difficult to determine the growth tendency given that there were only two time points used. More measured time points are required to better determine the change of psychopathic traits over time.

## Limitations

The findings from the current study should be considered along with the study’s limitations. First, participants in this study were predominantly recruited from southeast of China, so results may not generalize to other geographic areas or cultures. Second, the current study examined the LMI of CPTI scores in children from the general community. Future research should further evaluate the LMI of CPTI scores in clinical samples or referred youth. Finally, this study only examined the LMI of CPTI scores over a one-year time interval. Future study is needed to test the LMI of the CPTI over longer time intervals.

## Conclusion

The current study furthers our understanding of the longitudinal factorial invariance of CPTI scores in late-childhood. Overall, the CPTI that had strict longitudinal measurement invariance (i.e., equality of factor patterns, loadings, intercepts, and item uniqueness) across time in children aged 8 to 12 years old, could be enabled to investigate the development and variation of psychopathic traits in late childhood. Also, the results demonstrate that, at least in children aged 8 to 12 years old, changes in CPTI scores across time can be attributed to actual changes in the child’s psychopathic personality.

## Supporting information

S1 File CPTI data. (XLS)
